# Monte Carlo simulation of the Spearman-Kaerber TCID50

**DOI:** 10.1186/2043-9113-2-5

**Published:** 2012-02-13

**Authors:** Niels H Wulff, Maria Tzatzaris, Philip J Young

**Affiliations:** 1 Bavarian Nordic GmbH, Fraunhoferstrasse 13, D-82152 Martinsried, Germany

**Keywords:** TCID50, Spearman-Kaerber, pfu, Euler's constant, ANOVA, Monte Carlo simulation

## Abstract

**Background:**

In the biological sciences the TCID50 (median tissue culture infective dose) assay is often used to determine the strength of a virus.

**Methods:**

When the so-called Spearman-Kaerber calculation is used, the ratio between the pfu (the number of plaque forming units, the effective number of virus particles) and the TCID50, theoretically approaches a simple function of Eulers constant. Further, the standard deviation of the logarithm of the TCID50 approaches a simple function of the dilution factor and the number of wells used for determining the ratios in the assay. However, these theoretical calculations assume that the dilutions of the assay are independent, and in practice this is not completely correct. The assay was simulated using Monte Carlo techniques.

**Results:**

Our simulation studies show that the theoretical results actually hold true for practical implementations of the assay. Furthermore, the simulation studies show that the distribution of the (the log of) TCID50, although discrete in nature, has a close relationship to the normal distribution.

**Conclusion:**

The pfu is proportional to the TCID50 titre with a factor of about 0.56 when using the Spearman-Kaerber calculation method. The normal distribution can be used for statistical inferences and ANOVA on the (the log of) TCID50 values is meaningful with group sizes of 5 and above.

## 1. Introduction

In the TCID50 assay the dilution where there is a 50% chance that one or more cells are infected, is estimated. The Spearman-Kaerber calculation method is often used to accomplish this estimate. The method was inspired by the articles of Spearman [1] and Kaerber [2] and is widely used by biologists (see Additional File 1 Appendix, Section 1.1). Finney [3] actually recommends the Spearman-Kaerber method over the method of Reed and Muench [4]. The Spearman-Kaerber method is also recommended by FAO on their web-site [5]. It is well known that this dilution estimate does not directly give the pfu, but rather a number that is proportional to the pfu. In the article by Bryan [6], the author finds that the pfu/TCID50 ratio must be ln(2) ≈ 0.69. This is however only true if the TCID50 is calculated using a curve-fit of the theoretical dilution curve (see Additional File 1 Appendix, Section 1.2): $P\left(x>0|K_{0},D\right)=1-e^{-\;\frac{K_{0}}{D}}$, in which case you could directly read off the pfu as the fitted parameter *K*_0 _and therefore would not need to calculate a TCID50 value anyway. Here, *x *is the number of virus particles found at dilution *D*, and *K*_0 _is the number of virus particles in the undiluted substrate, i.e. the pfu. One could argue that such a curve-fit is the more appropriate approach in calculating the pfu. However, the simplicity of the Spearman-Kaerber calculation makes it the method of choice since it gives a number which is proportional to the pfu. When the Spearman-Kaerber method is used, the pfu/TCID50 ratio is about 20% lower than that estimated by Bryan, namely approximately 0.56. This value can be derived from the theoretical calculation in Govindarajulu [7] as *e*^-*γ*^, *γ *being Euler's constant 0.5772156649. The standard deviation of the natural logarithm of the TCID50 is found to be $\sqrt{\frac{\ln\left(D_{f}\right)\ln\left(2\right)}{n}}$, where *D_f _*is the dilution factor and *n *is the number of wells inspected per dilution step, ibid (see Additional File 1 Appendix, Sections 1.3 and 1.4). Thus, the aim of this paper is to show that the above theoretical calculations by Govindarajulu [7] is actually accurate in a practical setup of the TCID50 assay, where the dilutions are not completely independent. Further, we aim to show that common statistical methods, that assumes normal distributions, works well on the (log) titers produced by the TCID50 assay although these results are discrete in nature.

## 2. Methods

### 2.1 Practical implementation of the TCID50 assay

In the practical implementation here, the dilutions take place in a series of tubes. The following description of the assay uses 10 such tubes. Furthermore, it uses a 12 column by 8 row *micro titre plate *(MTP) and uses a factor 10 dilution for each dilution step (only the first 10 columns are used for the dilution steps, the two last columns are for control purposes). At the start, each tube contains 900 μl of cell culture media. In the first tube 100 μl of the test sample is added to the 900 μl cell culture media. Next, 100 μl is transferred from the first tube to the 2nd tube, then 100 μl is transferred from the 2^nd ^tube to the 3^rd ^tube and so on until the 10^th ^tube. There is now 900 μl fluid in the tubes 1 to 9 and 1000 μl in tube 10. The 8 wells in the first column of the MTP each receive 100 μl from the first tube. Similarly, the 8 wells in the second column of the MTP receive 100 μl from the second tube each etc. This means that the each well in the first column of the MTP contains (about) 1/10 of the infectious units in the test sample. Each well in the second column of the MTP contains (about) 1/100 of the infectious units in the test sample and so on across to the 10th column of the MTP where each well contains (about) 1/10^10 ^of the infectious units. In this manner each well in the first column of the MTP has 100 μl of the virus substrate diluted with a factor 10, each well in the second column of the MTP has 100 μl of the virus substrate diluted with a factor 100, etc. across to the 10^th ^column where each well in the column of the MTP has 100 μl of the virus substrate diluted with a factor 10^10^. Using this scheme, it is clear that the number of virus particles at each dilution is not completely independent: if the number of virus particles is larger than expected at some dilution step, then it is likely that the number of virus particles at the next dilution step will also be larger than expected. Similarly, if the number of virus particles is smaller than expected at some dilution step, then it is likely that the number of virus particles at the next dilution step will also be smaller than expected, i.e. there is a positive correlation between the dilution steps. The following Monte Carlo simulation shows that this, in fact, does not yield a ratio different from the theoretical pfu/TCID50 ratio above.

### 2.2 Monte Carlo simulation of the assay

The practical implementation using the above described scheme was precisely emulated using simulation software created by the author Niels Holger Wulff in the computer language C. The main algorithm is a routine that takes out a fraction *p *virus particles from *K*_0 _number of virus particles. The number of virus particles that is actually taken out, *K*_1_, is taken randomly from a binomial distribution:

$$P\left(K_{1}|K_{0}\right)=\left(\begin{array}{c} K_{0}\\ K_{1} \end{array}\right){\left(1-p\right)}^{\left(K_{0}-K_{1}\right)}p^{K_{1}}$$

We use the method called Von Neumann rejection to get and actual value, *K*_1_. For more details see Additional File 1 Appendix, Section 1.5. The random generator used is the routine RANMAR (based on work by George Marsaglia, Arif Zaman and Wai Wan Tsang) which is described in the article of James [8].

## 3. Results

For the dilution-10 assay (i.e. *D_f _*= 10) the average over 51 simulations with log10(*K*_0_) values of 3, 3.1, 3.2 ... 8 resulted in an average of: pfu/TCID50 = 0.5619 (SE = 0.0023). A dilution-2 assay (i.e. *D_f _*= 2) was also simulated (again with 51 log10(K0) values of 3, 3.1, 3.2 ... 8)-here the average was: pfu/TCID50 = 0.56135 (SE = 0.00019). These two results should be compared with the theoretical value of *e*^-*γ *^= 0.56146. The results indicate that even though the independence assumption is theoretically broken somewhat, the practical impact of this is quite small. It should be noted though, that due to the discrete nature of the Spearman-Kaerber calculation, the individual calculation of the pfu/TCID50 ratio will vary on the second decimal for the dilution-10 assay in a systematic way depending of the value on *K*_0 _for a fixed starting dilution. Thus, since we do not know the pfu (the *K*_0_) of a given virus substrate from the start, it normally only makes sense to state the ratio with two significant digits as 0.56 for the dilution-10 assay. For the dilution-2 assay, the ratio can be determined with one more digit as 0.561. This is similar to the finding in Finney [3] who finds that the Spearman-Kaerber TCID50 is not an unbiased estimate of the true underlying mean, *μ*, but rather depends on *the location of μ relative to the nearest *discrete dilution (p. 396). This small systematic variation also affects the standard deviation of the TCID50 values. For the dilution-10 assay, the average was found to be 0.194, but varies systematically between about 0.181 and 0.204. This should be compared with the theoretical value: $\sqrt{\frac{\ln\left(10\right)\ln\left(2\right)}{8}}/\ln\left(10\right)$ = 0.194 (we divide with ln(10) because the simulation calculations are done on a log10 scale). For the dilution-2 assay the average standard deviation was found to be 0.1061. Again, this should be compared with the theoretical value: $\sqrt{\frac{\ln\left(2\right)\ln\left(2\right)}{8}}/\ln\left(10\right)$ = 0.1064. Thus, for the standard deviation, the theoretical values are also very close to those obtained in the Monte Carlo simulation.

### 3.1 Distribution of the TCID50 values

The TCID50 values calculated using the Spearman-Kaerber method are drawn from a discrete distribution. For the practical implementation we used the base-10 logarithm and a plate with 8 rows yielding a discrete spacing of 1/8 for the dilution-10 assay.

#### 3.1.1 The simulated values

**Figure 1:**
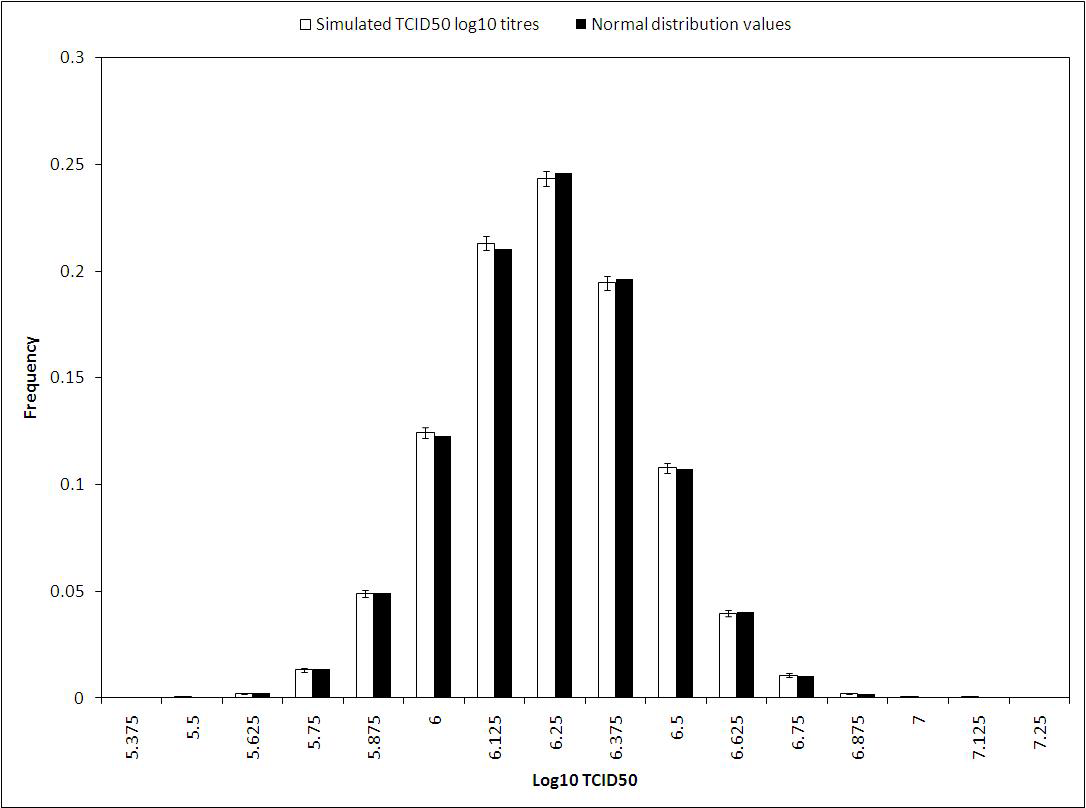
**Distribution of 60000 log10 titres with an average of 6.24 and a SD of 0.20 compared with the normal distribution with the same average and SD (both sets of bars have been normalized)**. The error bars are 95% Clopper Pearson CI.

Figure 1 shows the distribution of 60000 simulated log10(TCID50) values from a dilution-10 assay compared with the normal distribution.

It is obvious that the discrete distribution of the simulated data can be described using the value of the normal distribution at the discrete x-values. The discrete titre values from the Spearman Kaerber calculation will be of the form: *kδ *where *k *is a positive integer and *δ *is the distance between two discrete measurements. Due to the close connection to the normal distribution, a good approximation for the probability of the log10 titre being smaller than or equal to a certain discrete value *kδ *yields:

$$P\left(\text{TCID50}\leq k\delta \right)\approx \int_{\;-\infty }^{\;\left(k+0.5\right)\delta }dx\frac{1}{\sqrt{2\pi }\sigma }\exp\left(-\frac{{\left(x-\mu \right)}^{2}}{2\sigma^{2}}\right)$$

For an SD of about 0.2 the maximal difference from the true accumulated sum is only about 0.004 for a dilution-10 assay. The rationale behind this approximation simply comes from the so-called midpoint rule for numerical integration.

#### 3.1.2 Values from actual measurements

**Figure 2:**
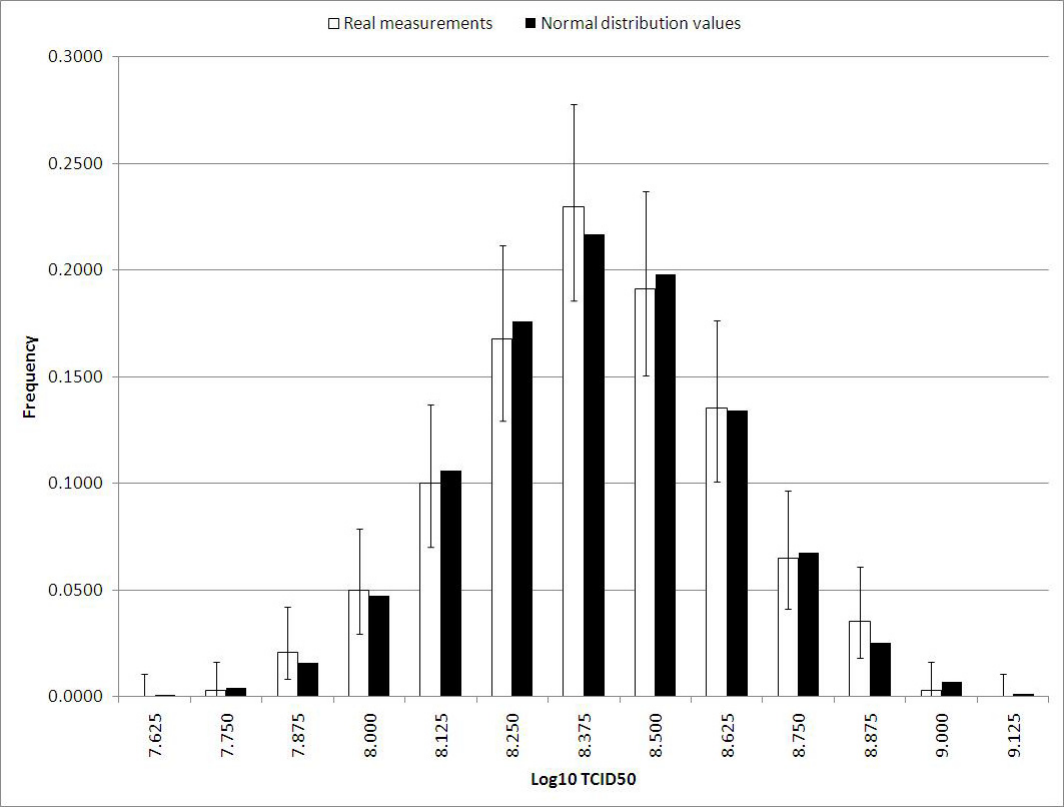
**Distribution of 340 measured log10 titres with an average of 8.400 and a SD of 0.229 compared with a normal distribution with the same average and SD (both sets of bars have been normalized)**. The error bars are 95% Clopper Pearson CI.

Figure 2 illustrates the distribution of 340 measurements of the control virus generated at Bavarian Nordic. The measurements are from the period: 16-Mar-2011 to 22-Jun-2011. The data behind the histograms in Figure 1 and 2 really are discrete, and a test for normality is therefore not appropriate. However, there is close resemblance to the values of the normal density function (evaluated at the discrete values that are the possible outcome of the Spearman-Kaerber algotithm): the frequencies from the normal density function are within the 95% Clopper Pearson confidence interval in all cases for the actual measured TCID50 values in Figure 2 and in all but one case (TCID50 = 5.625) for the simulated values in Figure 1.

### 3.2 ANOVA on TCID50 values

The close connection to the normal distribution suggests that ANOVA analysis is sensible between groups of discrete log10 titers. To demonstrate this we performed a number of homoscedastic *t*-tests on identically distributed groups made up of the 60000 simulated TCID50 titer values in Figure 1. The p-value calculation for a homoscedastic t-test is mathematically identical with the p-value from a one-way ANOVA on two groups.

The 60000 simulated TCID50 titers were divided into:

1) 6000 group pairs of 5 TCID50 values (2*5*6000 = 60000)

2) 5000 group pairs of 6 TCID50 values (2*6*5000 = 60000)

3) 3333 group pairs of 9 TCID50 values (2*9*3333 = 59994)

4) 2500 group pairs of 12 TCID50 values (2*12*2500 = 60000)

If the group size is too small one can easily encounter a situation where the values in each of the two groups are identical (and hence a t-test/ANOVA is meaningless). For a group size of 5 this probability is between 3 and 4 per million (given an SD of 0.194) and therefore considered negligible for the p-value calculation below (for group sizes lower than 5 one should consider other statistical methods than t-test/ANOVA).

For identically normal distributed groups one will expect that the ANOVA or the homoscedastic *t*-test have a *p*-value below 0.05 for about 5% of the group pairs (given independence) and similarly that about 1% have a *p*-value below 0.01. The results are summarized in Table 1. From this table it is clear that the *t*-test creates realistic *p*-values for all the four group sizes even though the numbers do not come from a normal distribution. This indicates that an ANOVA will give realistic *p*-values for group sizes down to 5 discrete log10 titre values for a dilution-10 assay.

**Table 1:** *t*-tests of groups of group size 5, 6, 9 and 12

Number of *t*-test's	6000	5000	3333	2500
Group size	5	6	9	12

Number of p-values less than 0.01	62	44	31	32
Percent p-values less than 0.01	1.0%	0.9%	0.9%	1.3%
Lower 95% Clopper Pearson CI	0.8%	0.6%	0.6%	0.9%
Upper 95% Clopper Pearson CI	1.3%	1.2%	1.3%	1.8%

Number of p-values less than 0.05	303	234	161	130
Percent p-values less than 0.05	5.1%	4.7%	4.8%	5.2%
Lower 95% Clopper Pearson CI	4.5%	4.1%	4.1%	4.4%
Upper 95% Clopper Pearson CI	5.6%	5.3%	5.6%	6.1%

## 4. Discussion

In this paper we have only studied the ideal situation, where the dilutions are completely precise and the wells are flawlessly "scored" as positive or negative. Naturally, this will not be the case in the real world where there will be both dilution errors and scoring errors. Furthermore, in the real world there are also day-to-day variations originating from unknown sources, and usually one will see that titres of the same material tend to be a little higher in some periods and a little lower in other periods. Thus, for real experiments the standard deviation naturally tends to be larger than the lower bounds described here. Thus, it is not surprising that the standard deviation of the 340 real data from the period: 16-Mar-2011 to 22-Jun-2011 in Figure 2, is larger than 0.194, namely 0.229.

In addition, we have dealt with the dilution-2 assay in the simulation study as if it was unproblematic to implement. Unfortunately, this is not the case. The practical problem here is the number of dilutions needed between the dilution where all the wells are positive and the dilution where all the wells are negative, call it the *drop length*. In order to make an acceptable calculation, the first column of the plate must have only positive wells and the last plate must have only negative wells. Furthermore, control wells are also required-in our implementation we use two columns for controls-leaving only 8 columns to encompass the drop. The simulation study actually showed that about 10% of all simulated dilution-2 experiments had a drop length above 8. In addition, it is not possible to pre-dilute the sample so that the first dilution that appears on the plate is the last dilution where all wells are positive (even if you have some prior knowledge of the titre). Thus, either two plates or one bigger plate is needed e.g. a 384 well plate (16 rows by 24 columns). Both solutions will be technically difficult for a laboratory-technician (for the bigger plate you will probably need a robot), potentially raising the variation of the assay. Note, that just 3 independent measurements with a dilution-10 assay on three 96-well MTP plates yields a theoretical lower bound of $0.194/\sqrt{3}\approx 0.112$, which is very close to the 8 row dilution-2 assay precision of 0.106. Although one could make use of the extra rows (theoretically this would increase the precision to a standard deviation of about $0.106/\sqrt{2}\approx 0.075$) it is questionable whether this would really be the case for a real implementation since the assay is technically complicated to perform and therefore error prone.

## 5. Conclusion

Monte Carlo simulation shows that in the practical implementation of the TCID50 assay, described in Section 2, the pfu is proportional to the TCID50 titre with a factor of about 0.56 when using the Spearman-Kaerber calculation method. This factor is the same as the theoretically calculated factor of *e*^-*γ*^, *γ *being Euler's constant 0.5772156649. The simulation further shows that theoretically calculated assay standard deviation of the log10 TCID50 values ($\sqrt{\frac{\ln\left(D_{f}\right)\ln\left(2\right)}{n}}/\ln\left(10\right)$) is close to the one calculated by the simulation. Although discrete in nature, the log of the TCID50 titre has a close relation to the normal distribution which can be used for statistical inferences. Finally, ANOVA seems to be meaningful comparing groups of log-titre results when the group sizes are 5 or above.

**Table 2:** Example of a dilution series

Log10(*D*)	1	2	3	4	5	6	7	8	9	10
Fraction of wells with positive response	1.000	1.000	1.000	1.000	1.000	1.000	0.875	0.125	0.000	0.000

**Figure 3:**
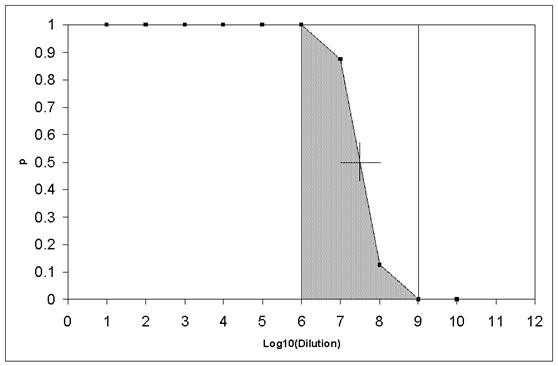
**The dilution curve for the example in Section 1.1 of the Appendix in the additional file ****1**.

## List of abbreviations

ANOVA: Analysis of Variance; MTP: Micro Titre Plate; pfu: the number of plaque forming units; TCID50: Median Tissue Culture Infective Dose.

## Competing interests

The authors declare that they have no competing interests.

## Authors' contributions

NHW carried out the Monte Carlo simulations and wrote the article.

PHY reviewed the mathematical and statistical content of the article.

MTZ reviewed the biological and lab-technical content of the article.

All authors have read and approved the final manuscript.

## Supplementary Material

Additional file 1 **Appendix**. The Spearman-Kaerber calculation method (example), The theoretical dilution curve, The theoretical pfu/TCID50 ratio, The theoretical standard deviation of the Spearman-Kaerber calculation, The Monte Carlo simulation program (the take-out algorithm and the simulation procedure in pseudo code), [[7,9] and Figure 3]. Click here for file
