# Supplementary material for: Monte Carlo simulation of the Spearman-Kaerber TCID50
Source: J Clin Bioinforma. 2012 Feb 13;2:5. doi: 10.1186/2043-9113-2-5 (PMC3331834; doi:10.1186/2043-9113-2-5)
Supplement: Additional file 1 — Appendix. The Spearman-Kaerber calculation method (example), The theoretical dilution curve, The theoretical pfu/TCID50 ratio, The theoretical standard deviation of the Spearman-Kaerber calculation, The Monte Carlo simulation program (the take-out algorithm and the simulation procedure in pseudo code), [[7,9] and Figure 3]. [file 2043-9113-2-5-S1.DOC]

# Appendix

## The Spearman-Kaerber calculation method

For a dilution assay following the scheme described in Section 2, the result could look like the dilution series shown in Table 2. Due to the way the assay is done each well in column *d* on the MTP contains (approximately) 1/*D*^th^ of the virus particles in the test sample, where *D* = 10*^d^*. Thus, the fraction of positive wells, in column *d* of the MTP, measures the probability of one or more infectious virus particle at dilution *D*. The dilution curve for the above example is seen in Figure 3.

The y-axis of the dilution curve is the fraction of wells where one or more reactions are seen, i.e. the fraction of *positive* wells. The x-axis is the log10 of the dilution. In essence, the Spearman-Kaerber algorithm determines the area under the dilution curve from the value 0 to the dilution where no reactions are seen, i.e. where all wells are *negative*. When the curve is close to point symmetry around the point where the line *p* = 0.5 cuts the curve (indicated with a cross on the plot), this area is a good approximation of the x-value of the symmetry point, i.e. a good approximation of the dilution where there are 50% probability of seeing a positive well. In practice the area is estimated by calculating only the area from the highest log-dilution where all wells are positive to the lowest log-dilution where all wells are negative (indicated as the grey area on the plot). The log10-dilution where all wells are positive is then added to this area yielding the log10 of the TCID50. In the Spearman-Kaerber calculation the area is calculated using simply the trapezoidal formula.

Let:

- *d*_1_ = the log of the highest dilution where all the wells are positive
- *N* = the number of dilutions after *d*_1_ where some wells are still positive
- *D_f_* = the dilution factor
- *d_f_* = the log of the dilution factor, *D_f_*
- *p_i_* = the fraction of positive wells for dilution *i* ($1\leq i\leq N+1$). Note: *p*_1_ is the fraction of positive wells at log-dilution *d*_1_, hence *p*_1_=1 and *p_N_*_+1_=0.
- *V*_test_ = the volume of the test substrate

The TCID50 is given by:

$$\begin{matrix} \log\left(\frac{\text{TCID}_{50}}{\text{Volume unit}}\right)=\text{Area}+d_{1}-\log\left(V_{\text{test}}\right)=\frac{d_{f}}{2}p_{1}+d_{f}\sum_{i=2}^{N} p_{i}+\frac{d_{f}}{2}p_{N+1}+d_{1}-\log\left(V_{\text{test}}\right) \\ =\frac{d_{f}}{2}+d_{f}\sum_{i=2}^{N} p_{i}+d_{1}-\log\left(V_{\text{test}}\right) \end{matrix}$$

Equation 1 The Spearman-Kaerber calculation of the TCID50

If $V_{\text{test}}=0.1$ml then for the above example where$d_{f}=1$ and$d_{1}=6$ we get: $\log\left(\frac{\text{TCID}_{50}}{\text{ml}}\right)=\frac{d_{f}}{2}+d_{f}\sum_{i=2}^{N} p_{i}+d_{1}-\log\left(V_{\text{test}}\right)=\frac{1}{2}+1\times\left(0.875+0.125\right)+6-\left(-1\right)=8.5$

## The theoretical dilution curve

If a certain substrate contains *K*_0_ infectious particles and you take out 1/*D* of the sample, the probability that none of the infectious particles is taken out is: $\left(1-\frac{1}{D}\right)^{K_{0}}$meaning that the probability that one or more of the infectious particles are taken out is:

$$P\left(x>0|K_{0},D\right)=1-\left(1-\frac{1}{D}\right)^{K_{0}}$$

Equation 2 The probability of one or more virus particles at dilution *D*

For large dilutions, i.e. *D* >> 1, this leads to:

$$P\left(x>0|K_{0},D\right)=1-\left(1-\frac{1}{D}\right)^{K_{0}}=1-\exp\left[K_{0}\ln\left(1-\frac{1}{D}\right)\right]\approx1-e^{- \frac{K_{0}}{D}}$$

Equation 3 The probability of one or more virus particles at dilution *D* when *D* >> 1

This result is also what you would get from a Poisson distribution assumption$P(x>0)=1-f\left(x=0;\lambda=\frac{K_{0}}{D}\right)=1-\frac{\lambda^{0}e^{-\lambda}}{0!}=1-e^{-\frac{K_{0}}{D}}$. As mentioned above, the Spearman-Kaerber calculation calculates the logarithm of the TCID50 by estimating the integral of the dilution curve in a semi logarithmic plot, i.e. it estimates the integral:

$$\ln\left(\text{TCID50}\right)=\int_{ 0}^{ \infty} dt\left(1-e^{- \frac{K_{0}}{\exp\left(t\right)}}\right)\equiv\int_{ 0}^{ \infty} dt p\left(t\right)$$

, where $t=\ln\left(D\right)$ and $p\left(t\right)=1-e^{- \frac{K_{0}}{\exp\left(t\right)}}$

## The theoretical pfu/TCID50 ratio

Both the Spearman-Kaerber formula and the theoretical calculation of the logarithm of the TCID50 titre above assumes that the number of virus particles in the undiluted sample,$K_{0}$, is large, i.e. that$K_{0}>>1$ so that the probability of anything else than a 100% response at dilution *D*=1 is negligible. The calculation of the theoretical pfu/TCID50 ratio is then straightforward:

First we make a backwards substitution with $t=\ln\left(\frac{K_{0}}{x}\right)$:

$$\int_{ 0}^{ \infty} dt\left(1-\exp\left(-\frac{K_{0}}{\exp\left(t\right)}\right)\right)=\int_{ 0}^{ K_{0}} dx\frac{1}{x}\left(1-\exp\left(-x\right)\right)$$

Integrating by parts gives:

$$\begin{matrix} \ln\left(\text{TCID50}\right)=\int_{ 0}^{ K_{0}} dx\frac{1}{x}\left(1-\exp\left(-x\right)\right)=\left[\ln\left(x\right)\left(1-\exp\left(-x\right)\right)\right]_{0}^{K_{0}}-\int_{ 0}^{ K_{0}} dx\ln\left(x\right)\exp\left(-x\right) \\ =\ln\left(K_{0}\right)\left(1-\exp\left(-K_{0}\right)\right)-\int_{ 0}^{ K_{0}} dx\ln\left(x\right)\exp\left(-x\right)\approx\ln\left(K_{0}\right)+\gamma\end{matrix}$$

since$_{x}\underrightarrow{\lim}_{0_{+}}\ln\left(x\right)\left(1-\exp\left(-x\right)\right)=_{x}\underrightarrow{\lim}_{0_{+}}x\ln\left(x\right)=0$,$K_{0}>>1$ and$\int_{ 0}^{ \infty} dx\ln\left(x\right)\exp\left(-x\right)=-\gamma$, where $\gamma$ is the Euler constant: 0.5772156649.

Thus: $\frac{K_{0}}{\text{TCID50}}=e^{-\gamma}\approx0.56146$

See also Govindarajulu [7].

## The theoretical standard deviation of the Spearman-Kaerber calculation

The variation of the Spearman-Kaerber result in Equation 1 can be estimated:

$Var\left(\ln\left(\text{TCID}_{50}\right)\right)=d_{f}^{2}\sum_{i} \frac{p\left(t_{i}\right)\left(1-p\left(t_{i}\right)\right)}{n}$, where *n* is the number of wells in a column of the MTP and $t_{i}$ is the discrete log-dilution, $t_{i}=\ln\left(D_{f}^{i}\right)=i\ln\left(D_{f}\right)=id_{f}$ (where $d_{f}$here is the natural logarithm of the dilution factor $D_{f}$and *p* is the function defined in Section 1.2).

Now: $d_{f}\sum_{i} p\left(t_{i}\right)\left(1-p\left(t_{i}\right)\right)\approx\int_{ 0}^{ \infty} dt\left(1-e^{- \frac{K_{0}}{\exp\left(t\right)}}\right)e^{- \frac{K_{0}}{\exp\left(t\right)}}$, and backwards substitution with $u=e^{- \frac{K_{0}}{\exp\left(t\right)}}\Leftrightarrow\ln\left(u\right)=- \frac{K_{0}}{\exp\left(t\right)}\Rightarrow dt=-\frac{1}{u\ln\left(u\right)}du$ yields:

$Var\left(\ln\left(\text{TCID}_{50}\right)\right)=\frac{d_{f}}{n}\int_{ 0}^{ \infty} dt\left(1-e^{- \frac{K_{0}}{\exp\left(t\right)}}\right)e^{- \frac{K_{0}}{\exp\left(t\right)}}=-\frac{d_{f}}{n}\int_{ 0}^{ 1} du\frac{1-u}{\ln\left(u\right)}=\frac{d_{f}\ln\left(2\right)}{n}$ , again assuming that$K_{0}>>1$. Thus, the standard deviation of the natural logarithm of the TCID50 becomes: $SD\left(\ln\left(\text{TCID}_{50}\right)\right)=\sqrt{\frac{\ln(D_{f})\ln(2)}{n}}$.

See also Govindarajulu [7].

## The Monte Carlo simulation program

The heart of the simulation program is a routine that takes out a certain fraction of infectious virus particles from a pool of particles. This procedure is described in detail below.

### *The probability of taking K_i +1_ infectious units from K_i_ infectious units*

The sample contains *N* units or “fluid particles” where *K*_0_ of these are infectious virus particles. When diluting the sample we take out a fraction, *p* (=1/(dilution_factor)), of the *N* particles. Statistically, the infectious virus particles that are taken out are found by going through the *K*_0_ infectious virus particles and assign each of them to the group that is taken out with a probability of *p*. This yields a binomial distribution:

$$P\left(K_{1}|K_{0}\right)=\left(\begin{matrix} K_{0} \\ K_{1} \end{matrix}\right)\left(1-p\right)^{\left(K_{0}-K_{1}\right)}p^{K_{1}}$$

Similarly, the probability of *K_i+_*_1_ infectious particles in dilution step *i*+1 given *K_i_* infectious particles in previous dilution step is calculated:

$$P\left(K_{i+1}|K_{i}\right)=\left(\begin{matrix} K_{i} \\ K_{i+1} \end{matrix}\right)\left(1-p\right)^{\left(K_{i}-K_{i+1}\right)}p^{K_{i+1}}$$

Equation 4

In practice, this formula is used the following way:

1. When *K_i_* is larger than 10^8^, *K_i+1_* = *pK_i_* because the relative standard deviation of the taken fraction is very small (less than 0.00005 (= worst case = $\sqrt{0.5\left(1-0.5\right)10^{8}}/10^{8}$).
2. When *K_i_* is 10^8^ or less, the distribution is calculated between the *non-trivial limits* defined as: m±4SD, i.e. the distribution is calculated from the nearest integer to $pK_{i}-4\sqrt{p\left(1-p\right)K_{i}}$ to the nearest integer to $pK_{i}+4\sqrt{p\left(1-p\right)K_{i}}$. Naturally, both integers must belong to the interval [0; *K_i_*]. Thus, if $pK_{i}-4\sqrt{p\left(1-p\right)K_{i}}<0$, the lower non-trivial limit is set to 0 and if $pK_{i}+4\sqrt{p\left(1-p\right)K_{i}}>K_{i}$, the upper trivial limit is et to $K_{i}$. Outside the non-trivial limits the distribution is considered to be 0.
3. The number of infectious particles in the next dilution step, *K_i+_*_1_, is then assigned a random value using Von Neumann rejection: The discrete *x*-values are chosen from a uniform distribution between the non-trivial limits, and the (continuous) *y*-values are chosen from a uniform distribution between 0 and the max of the distribution in Equation 4. Call the distribution *f*(*x*). If *y* < *f*(*x*) then the *x*-value is accepted otherwise it is rejected and another pair (*x*, *y*) is chosen. This is repeated until an *x*-value is accepted. The accepted *x*-values will be distributed according to *f*(*x*). The maximum of a binomial distribution is found at either $\left\lfloor Np\right\rfloor$ or $\left\lfloor Np\right\rfloor$+1, where $\left\lfloor x\right\rfloor$ is the symbol for the floor of *x*, i.e. largest integer lower than or equal to *x* – see e.g. Foster et al. [9].

The three points described above is implemented in a routine called take_fraction that takes an integer, *K*, and a floating point value, *p*, as arguments and returns another integer – the number of infectious virus particles taken out from the *K* inputted particles.

### *The simulation procedure in pseudo code*

Dilution factor = 10:

rows = 8

knext = Number_of_infectious_particles_in_sample

For c = 1 To columns ' in the simulation the plate columns was set to 40

p = 1 / dil_factor

kleft = take_fraction(knext, 1 - p) ' this is what is left in the cluster

' tube after taking out a fraction, p,

' for the next cluster tube

knext = knext - kleft ' rolling

For r = 1 To rows ' distribute on wells on the 8 MTP rows

p = 1 / (rows - r + 2) ' in row 1: 1/9 of 900µl, in 2: 1/8 of 800µl etc.

well(r) = take_fraction(kleft, p) 'the virus particles in well r

kleft = kleft - well(r) ' rolling

Next r

Next c

Dilution factor = 2:

rows = 4

' first set of cluster tubes

knext = Number_of_infectious_particles_in_sample

For c = 1 To columns ' in the simulation the plate columns was set to 40

p = 1 / dil_factor

kleft = take_fraction(knext, 1 - p) ' this is what is left in the cluster

' tube after taking out a fraction, p,

' for the next cluster tube

knext = knext - kleft ' rolling

For r = 1 To rows ' distribute on wells on the 4 MTP rows

p = 1 / (rows - r + 2) ' in row 1: 1/5 of 500µl, in 2: 1/4 of 400µl etc.

well(r) = take_fraction(kleft, p) 'the virus particles in well r

kleft = kleft - well(r) ' rolling

Next r

Next c

' second set of cluster tubes

knext = Number_of_infectious_particles_in_sample

For c = 1 To columns ' in the simulation the plate columns was set to 40

p = 1 / dil_factor

kleft = take_fraction(knext, 1 - p) ' this is what is left in the cluster

' tube after taking out a fraction, p,

' for the next cluster tube

knext = knext - kleft ' rolling

For r = 1 To rows ' distribute on wells on the next 4 MTP rows

p = 1 / (rows - r + 2) ' in row 1: 1/5 of 500µl, in 2: 1/4 of 400µl etc.

well(r + rows) = take_fraction(kleft, p) 'the virus particles in well r+rows

kleft = kleft - well(r + rows) ' rolling

Next r

Next c
